# INST OX‐05‐024: first line gemcitabine, oxaliplatin, and erlotinib for primary hepatocellular carcinoma and bile duct cancers: a multicenter Phase II trial

**DOI:** 10.1002/cam4.1138

**Published:** 2017-08-11

**Authors:** Yehuda Z. Patt, Waheed Murad, Mohammed H. Fekrazad, Ari D. Baron, Pranshu Bansal, Yanis Boumber, Kim Steinberg, Sang‐Joon Lee, Ed Bedrick, Ruofei Du, Fa Chyi Lee

**Affiliations:** ^1^ Division of hematology/oncology, Department of medicine University of New Mexico Comprehensive Cancer Center Albuquerque New Mexico; ^2^ University of California Riverside and Kaiser Permanente Riverside Moreno valley California; ^3^ City of Hope National Medical Center Durate California; ^4^ Pacific Hematology Oncology Associates San Francisco California; ^5^ Fox chase cancer center Philadelphia Pennsylvania; ^6^ Division of Epidemiology and Biostatistics Department of Medicine University of New Mexico Albuquerque New Mexico; ^7^ Cellitron Inc Product analysis division Incheon Korea

**Keywords:** Bile Duct Cancer, Erlotinib, Gemcitabine, HCC, Oxaliplatin

## Abstract

Hepatocellular Carcinoma (HCC) incidence is increasing in the USA. Gemcitabine (G) and oxaliplatin (O) are active in HCC and biliary duct cancer (BDC). Erlotinib (E) is an EGFR tyrosine kinase inhibitor (TKI) with known activity against both. We sought to evaluate the efficacy of the combination G+O+E. Patients with either of the two diagnosis were treated in a phase II trial. Simons 2 stage design was used. A disease‐control rate (DCR), complete response (CR) + partial response (PR)+ stable disease (SD) at 24 weeks of ≤20% and >40% (P0 and P1 of 0.2 and 0.4, respectively) were set as undesirable (null) and desirable results. 26 HCC and 7 BDC patients were accrued. In HCC, 1 PR, 10 SD, and 9 PDs were seen. DCR in HCC was 42%. Among seven (7) patients with BDC, one patient was not evaluable; one achieved a long lasting PR, and five patients had SD and DCR was 86%. Median overall survival (OS) times and progression‐free survivals (PFS) were 196 and 149 days in HCC and 238 days and not reached in BDC. PFS at 26 weeks in HCC was 41% and at 21 weeks in BDC was 60%. Grade 3 toxicities in >5% of patients were fatigue (12.9%), neutropenia (9.6%), thrombocytopenia (9.6%), and diarrhea (6.4%). G+O+E exceeded both preset P0a and P1 of the primary objective with a PFS of 41% at 26 weeks for HCC and preliminary BDC data may warrant further investigations.

## Introduction

The incidence of Hepatocellular Carcinoma (HCC) has been increasing worldwide and in the United States (US) 1. In the US this has been attributed to the increasing incidence of chronic hepatitis C viral infection which now affects approximately 2% of the US population, and an increase in the prevalence of alcoholic and nonalcoholic fatty liver disease (NAFLD) 2, 3. Gall bladder and biliary duct cancer (BDC), are relatively less common in US, but some of the highest rates in North America have been reported in American Indian and Mexican American population. These two groups together comprise> 50% of the population of New Mexico (NM), thus making the accrual for a relatively rare cancer slightly easier in New Mexico 4, 5. The combination of Gemcitabine and Oxaliplatin had been reported to have antitumor activity in both HCC and in BDC 6, 7. Additionally, EGFR overexpression and amplification have been shown in a majority of hepatocellular and biliary ductal carcinomas 8, 9. Erlotinib, an EGFR tyrosine kinase inhibitor, has demonstrated activity against HCC as well as BDC 10, 11. Therefore, this phase II trial of combination of gemcitabine, oxaliplatin plus erlotinib was launched in patients with HCC. Following the observation of antitumor activity in a patient who was initially misdiagnosed as having HCC and subsequently found to have BDC, it was decided to open this trial to patients with both hepatocellular carcinoma as well as biliary duct cancer, and an identical treatment regimen was used in both groups of patients.

The primary objective of this multicenter Phase II trial was to determine the rate of disease stability (% of patients with Complete Response [CR], Partial Response [PR], and Stable Disease [SD] at 24 weeks following the initiation of protocol treatment). Secondary objectives included: antitumor response rate, (the rate of CR and PR among both HCC and BDC patients), determination of median overall survival (OS) time and assessment of treatment‐related toxicities.

## Methods

### Study design and participants

In this prospective, open label, phase II trial patients were enrolled at the University of New Mexico Cancer Center (UNMCC) in Albuquerque, NM and at the California Pacific Medical Center (CPMC) in San Francisco, CA. Eligibility criteria for participation in this protocol required CT or MRI imaging, confirming measurable HCC or BDC (Table 1). For patients with liver cirrhosis due to hepatitis C or B viral infection or with alcoholic liver cirrhosis, presence of serum alpha‐fetoprotein (AFP) of >400 Nanograms per milliliter and imaging studies (CT scan or MRI) findings diagnostic of HCC were sufficient to confirm an HCC diagnosis. Histologic confirmation of the tumor was not a prerequisite for registration on this phase II trial for patients meeting both AFP and imaging criteria.

**Table 1:** Patient diagnoses and demographic data

Diagnosis		HCC	BDC	All		
Total		26	7	33		
Site	UNMCC	14	4	18		
	CPMC	12	3	15		
Age (yrs)	Median	57				
	Range	43–77				
Gender	Male	26				
	Female	7				
Ethnicity	White	Hispanic	Asian	AI	AA	Unknown
	14	9	6	2	2	1

HCC, Hepatocellular Carcinoma; UNMCC, University of New Mexico Cancer Center; CPMC, California Pacific Medical Center, San‐Francisco, CA. AI, American Indian; AA, African American.

In contrast, for patients with biliary duct cancers, both radiologically measurable and histologically confirmed diagnosis of BDC were prerequisites. In addition, a decreasing serum bilirubin to less than 3.5 milligrams per deciliter (dL) following decompression of obstructive jaundice was mandated.

Among patients with HCC, a Child‐Pugh class A or B, adequate renal function, a performance status of ECOG 2 or less, and a life expectancy of greater than 12 weeks were required for registration on the HCC arm provided the platelet count exceeded 100,000 per *μ*L (microliter). Among patients with BDC, adequate bone marrow function, and adequate renal and hepatic reserves were also prerequisites. Baseline laboratory values have been mentioned in Table 2. At the time of inclusion in the study patients had advanced HCC not eligible for resection or loco regional therapy or metastatic HCC. Of the 31 HCC patients one had prior transarterial embolization performed and one had prior treatment with radiofrequency ablation the rest 29 were treatment niave, similarly all seven BDC patients were treatment naïve locally advanced (nonresectable) or metastatic disease patients.

**Table 2:** Baseline laboratory findings

Baseline laboratory values	Value	Range
Median platelets (x 10^3^/mm^3^)	193	99–668
Albumin (gm/dL)	3.5	2.3–4.8
Total bilirubin (mg/dL)	0.8	0.2–1.7

Eighteen patients were entered at the UNMCC and 15 at CPMC. The total number of treatment cycles given was 118, with a median number of treatment cycles of 3 and a range of 1–27.

Among 39 patients registered on this trial, there were 14 Caucasians, nine Hispanics, six Asians, two Native Americans, and two African Americans, whereas ethnicity information was not available for one patient. Factors contributing to chronic liver disease are summarized in Table 3. Thus, liver cirrhosis due to alcohol exposure, HCV/HBV infection, or combinations of those were contributing factors in both HCC and BDC.

**Table 3:** Patients' predisposing and performance factors

Predisposing factors	HCC	BDC	Total
Clinical cirrhosis	9	2	11
Child‐pugh class A			9
Child‐pugh class B‐7			2
ETOH	13	2	15
HBV+	7		7
HCV+	14		14
HCV+/HBV+	3^a^		3
Prior treatments			
None	24	7	31
TACE	1		1
RFA	1		1

HCC, Hepatocellular Carcinoma; ETOH, Ethanol.

a Included among the 14 HCV + patients.

### Procedures

Gemcitabine at a dose of 1000 mg/m^2^ was given on day one, whereas oxaliplatin at a dose of 100 mg/m^2^ was given on day two. In addition, erlotinib (Tarceva^®^) was given daily at a dose of 150 milligrams PO. Reduced dose levels (minus 1 and minus 2) were as follows: 750 mg and 75 mg/m^2^ and 650 mg and 65 mg per m^2^ of gemcitabine and oxaliplatin, respectively. Dose modifications for erlotinib were as follows: 100 milligrams daily at dose level minus 1, 75 milligrams daily at dose level minus 2, and 50 mg daily at dose level minus 3.

Gemcitabine plus oxaliplatin administrations were repeated every 15 days, and two repetitions constituted one treatment cycle. All patients with HCC with Child‐Pugh class B liver cirrhosis were started at dose level minus 1, or 100 milligrams daily. The dose of erlotinib was adjusted according to degree of skin toxicity. All BDC patients were also started on erlotinib at 100 milligrams daily (dose level ‐1). Simultaneous administration of erlotinib along with warfarin type anticoagulants was disallowed following an initial fatal adverse event (patient 1). The treatment dosing plan is summarized in Table 4 and 5.

**Table 4:** Treatment plan

Agent	Starting dose (mg/m²)	Adjustments ‐1	Adjustments ‐2	Adjustments ‐3
Gemcitabine	1000	750	600	500
Oxaliplatin	100	75	60	50
Tarceva Daily (mg)	150	100	75	50

Treatment Days: 1, 15, 29, 43, etc.

Day 1 &15 treatment = 1 Cycle, Cycles were repeated every 4 weeks.

**Table 5:** Treatment summary

Treatment summary	Results
Number of patients	33
Total number of cycles	118
Median	3
Range	1–27

### Outcomes

The primary objective of this multicenter Phase II trial was to determine the rate of disease stability (% of patients with Complete Response [CR], Partial Response [PR], and Stable Disease [SD] at 24 weeks following the initiation of protocol treatment). Secondary objectives included: antitumor response rate, (the rate of CR and PR among both HCC and BDC patients), determination of median overall survival (OS) time and assessment of treatment‐related toxicities.

### Statistical design and analysis

The widely applied Simon's Two‐Stage Optimal design was chosen for this Phase II study. Disease control rate (DCR: CR+PR+SD) at 24 weeks of the treatment serves as the primary endpoint to test the efficacy of the combination of gemcitabine, oxaliplatin and erlotinib on the defined target population. A DCR rate greater than or equal to 40% would be considered worth of further investigation, whereas a DCR of less than or equal to 20% would be considered unacceptable. The number of final evaluable patients is 25, with which we are able to achieve >80% power for the test, at the significance level of 0.1.

The trial was successful and completed at the end of the 2nd stage. A confidence interval of the estimated DCR rate was calculated by Wilson method without continuity correction 12. The survival function was estimated by Kaplan–Meier approach, with confidence intervals constructed by the log cumulative hazard transformation method (i.e., log‐log transformation). All the analysis was done using SPSS version 15.0.

### Response data

**Figure 1:**
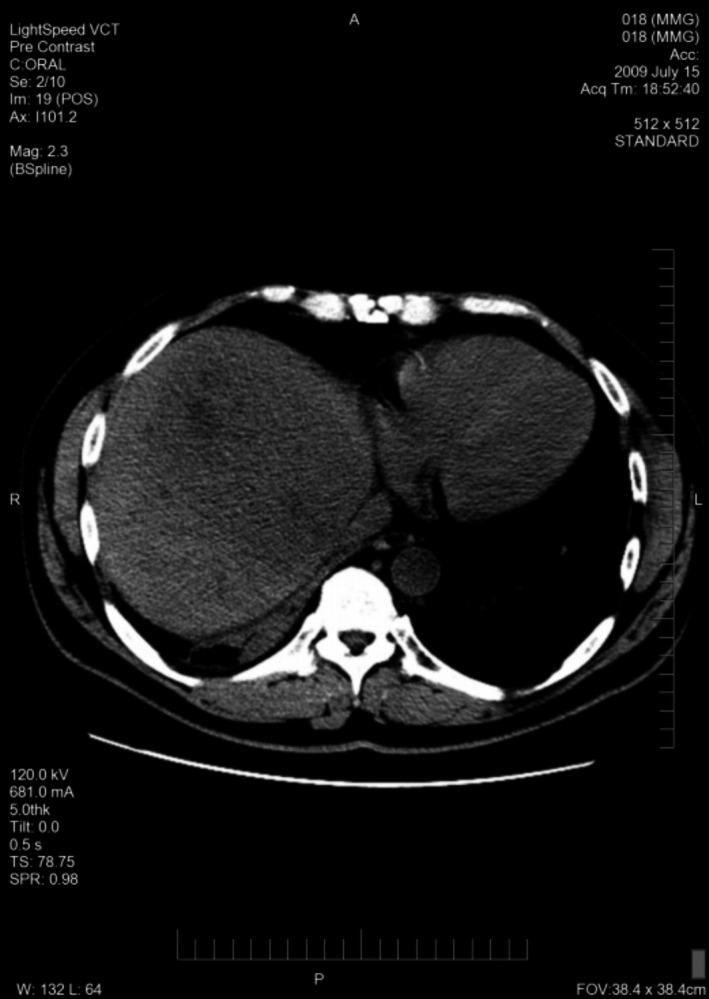
Patient 1 with Hepatocellular Carcinoma (HCC)—Baseline CT images without contrast, tumor at baseline (prior to trial enrollment) was measured at least 13.3 X 9.5 cm in segments 8 and 4a.

**Figure 2:**
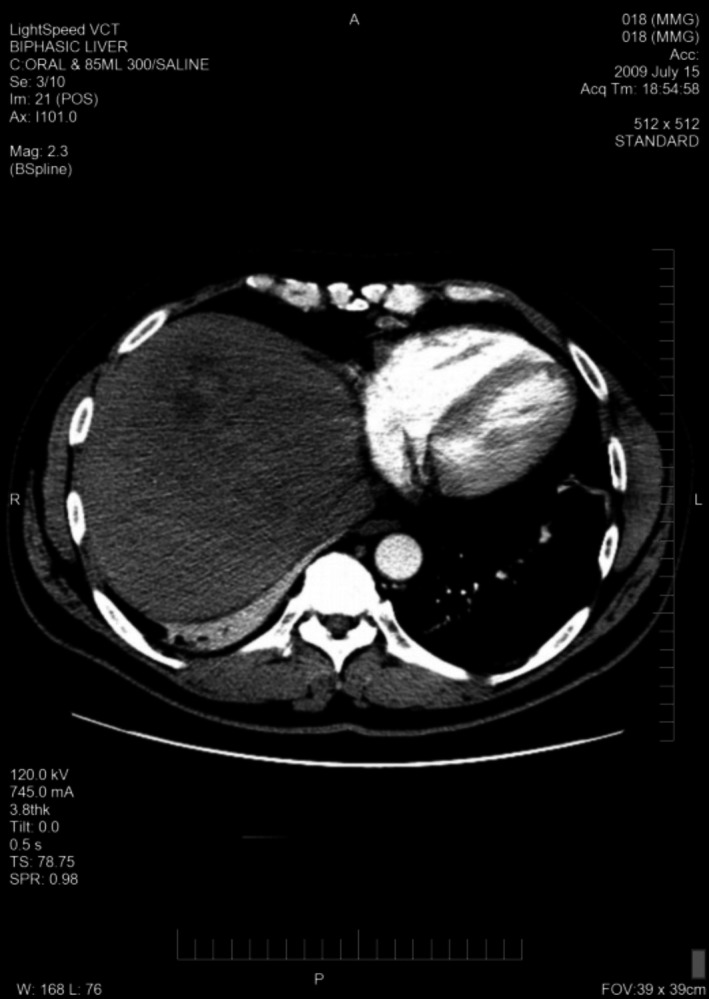
Patient 1 with Hepatocellular Carcinoma (HCC)—Baseline CT images—arterial phase.

**Figure 3:**
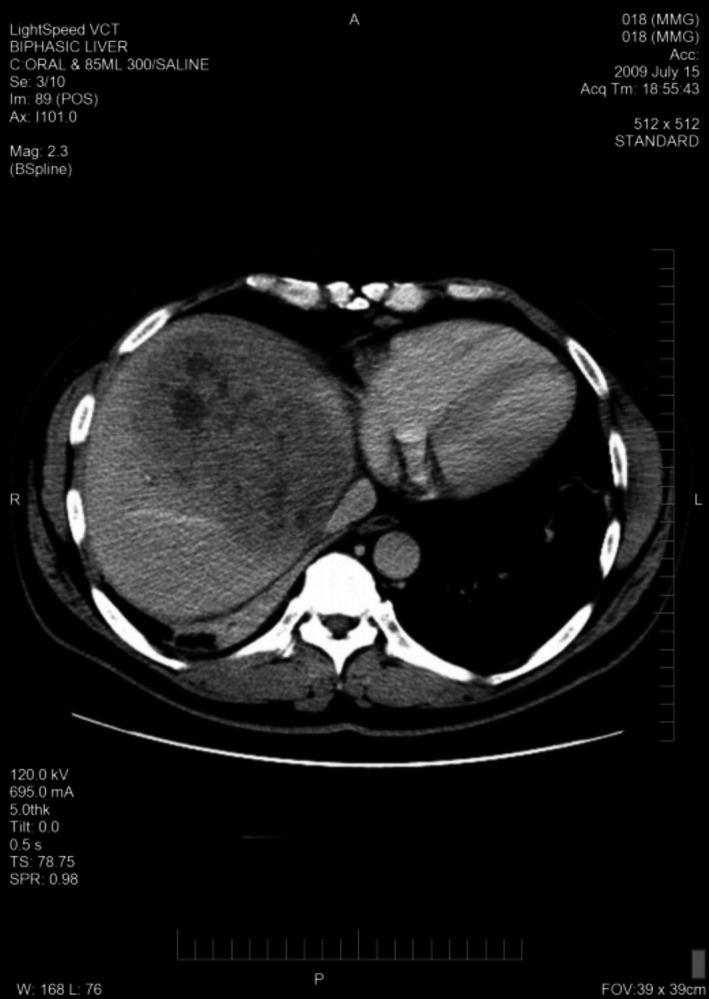
Patient 1 with Hepatocellular Carcinoma (HCC)—Baseline CT images—venous phase.

**Figure 4:**
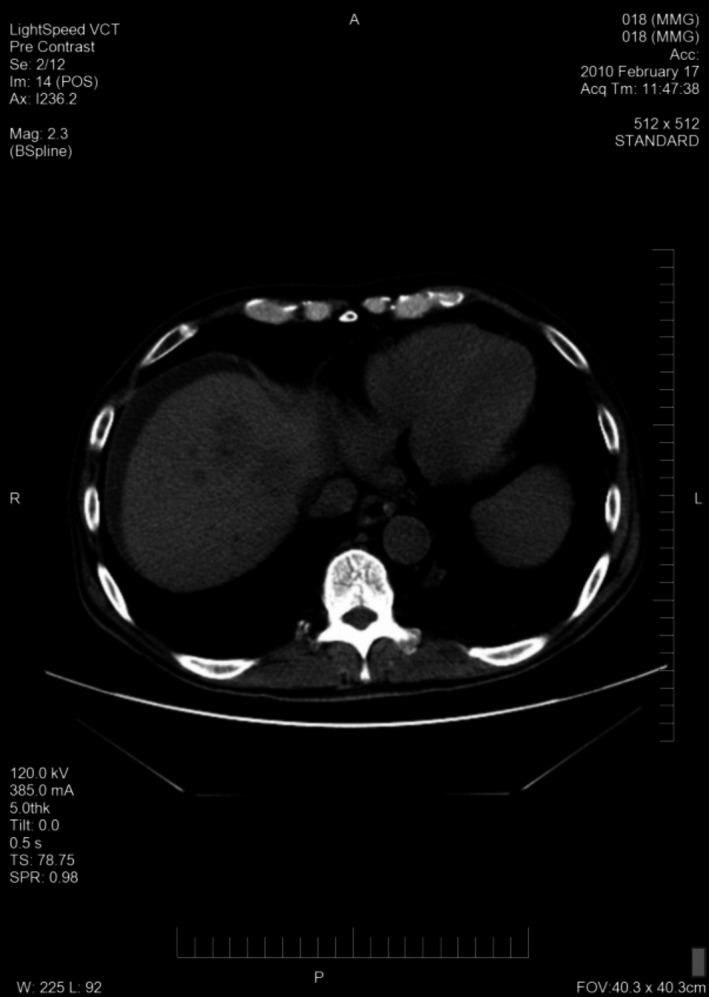
6‐month follow‐up CT image ( without contrast) of patient 1 with Hepatocellular Carcinoma (HCC) the maximal tumor diameter was 6.6 x 2.6 cm showing a good PR on trial.

**Figure 5:**
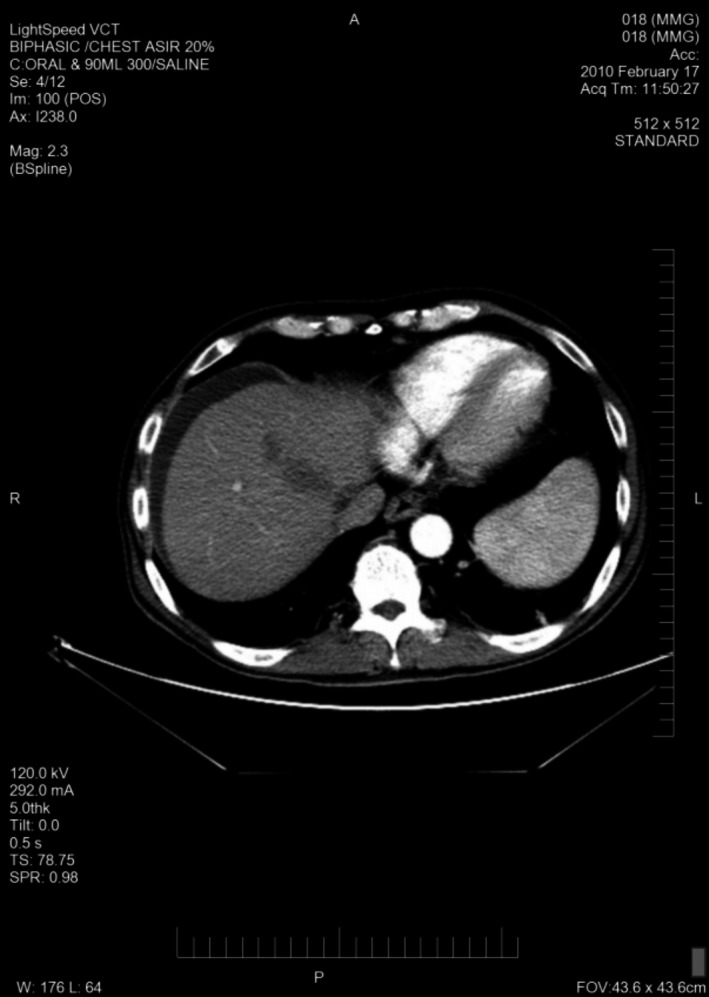
Patient 1 with Hepatocellular Carcinoma (HCC), 6‐month follow‐up CT arterial phase.

**Figure 6:**
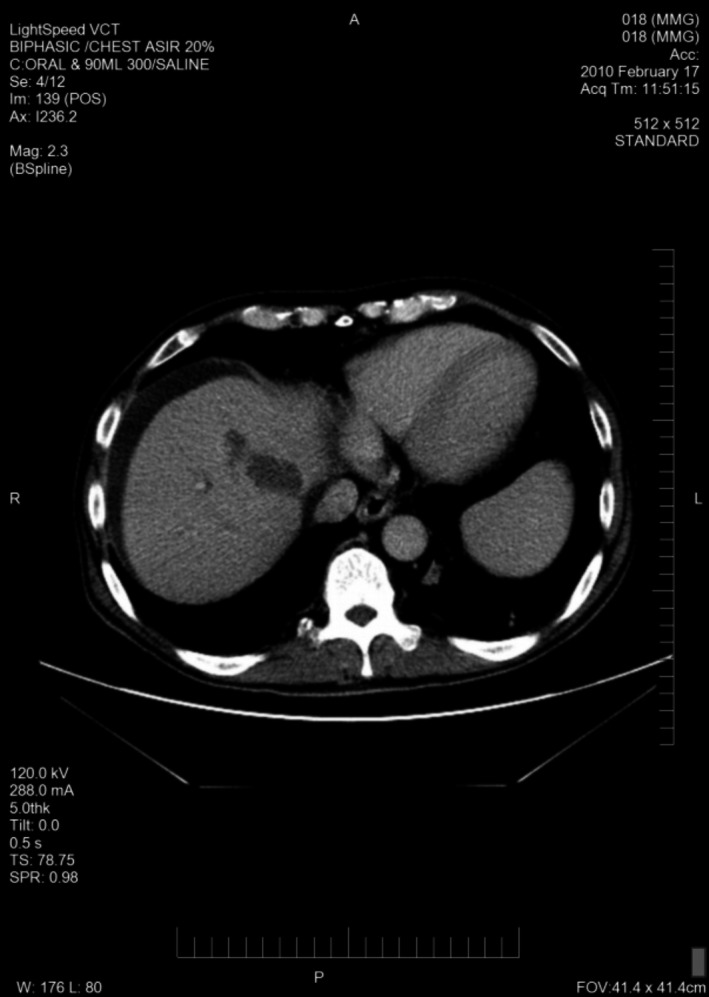
Patient 1 with Hepatocellular Carcinoma (HCC), 6‐month follow‐up CT portal venous phase.

**Figure 7:**
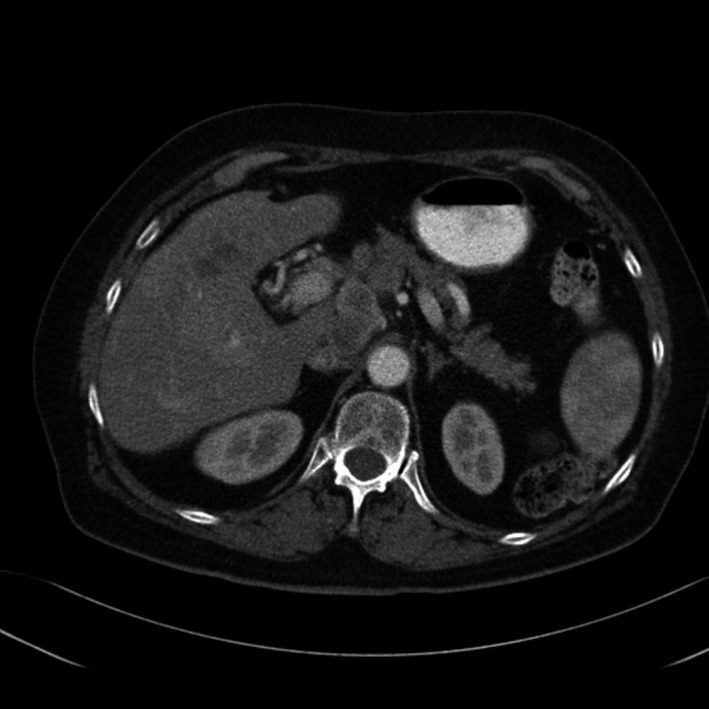
Patient 2—Baseline CT image arterial phase. This patient's the tumor at baseline (prior to trial enrollment) was measured at 3.1 X 4.2 cm in segments 4b and 5.

**Figure 8:**
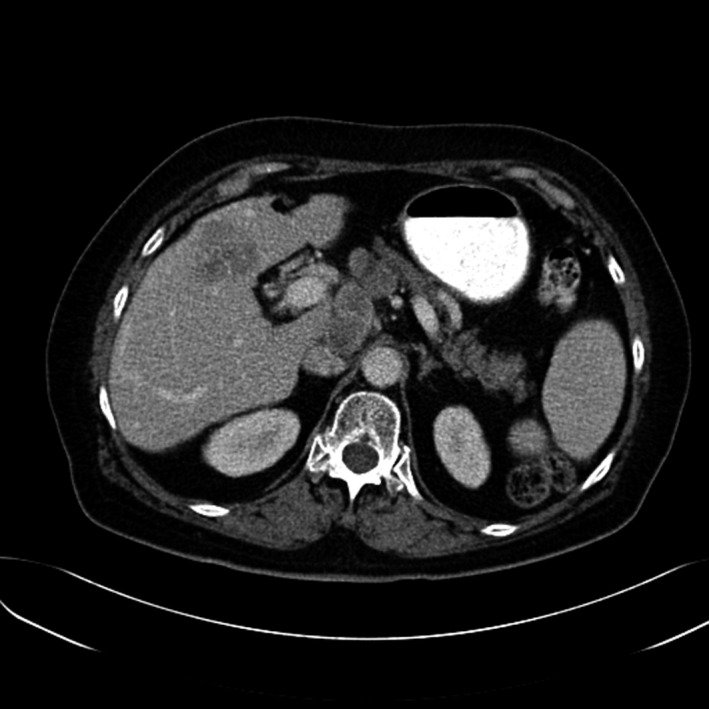
Patient 2. Baseline CT image portal venous phase.

**Figure 9:**
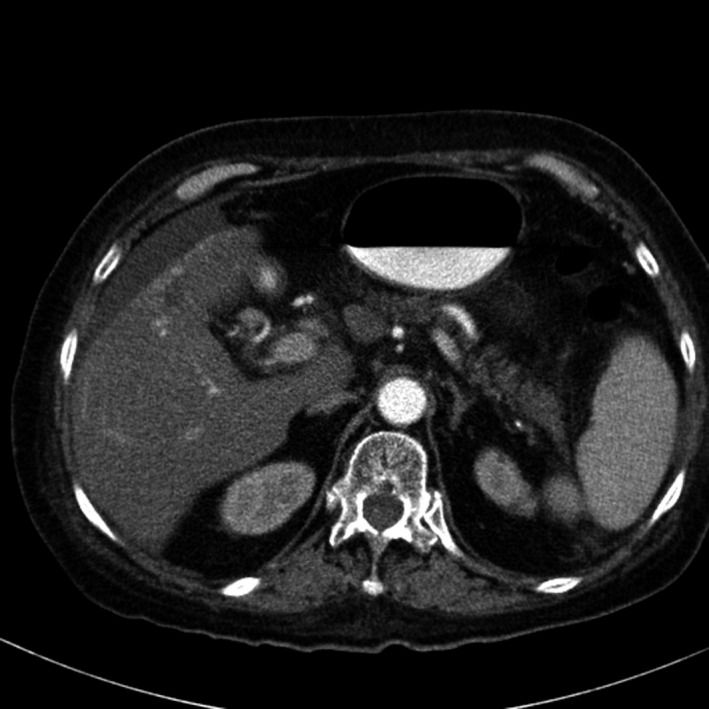
Patient 2. 2.5‐month follow‐up CT image arterial phase, the maximal tumor diameter was 2.9 x 2.3 cm, this patient had worsening complications of cirrhosis as well as can be seen by evolving ascites although the tumor was showing some response.

**Figure 10:**
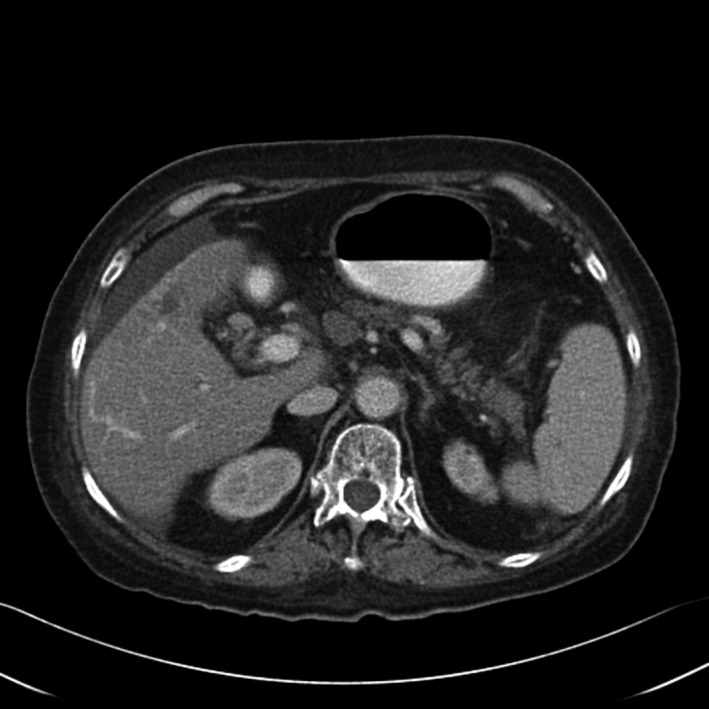
Patient 2 follow‐up CT image venous phase.

A total of 33 patients were enrolled (26 with HCC and seven with BDC). Among these patients, response to treatment could not be determined in five patients (four with HCC and one with BDC) due to lack of follow‐up CT scans. Therefore, 28 evaluable patients were included in the response analyses. Among 26 HCC patients, one had a PR (CT images, Figs. 1, 2, 3, 4, 5, 6), and 10 had SD (CT images, Figs. 7, 8, 9, 10) for a HCC DCR of 42% (11/26), whereas 11 patients had PD (progressive disease). Among seven (7) patients with BDC, one patient was not evaluable; one achieved a long lasting PR, and five patients had SD, for a BDC disease control rate (DCR) of 86% (6/7) (summarized in Table 6).

**Table 6:** Response summary (All patients)

Response	HCC	BDC	Total (HCC+BDC)
IE*	4	1	5
PR	1	1	2
SD	10	5	15
PD	11	0	11
Disease control rate, DCR	11/26 42% (95% CI: 25.6–61%)	6/7 86% (95% CI: 48.7–97.4%)	17/33 51.5% (95%: CI 35.2–67.5%)

HCC, Hepatocellular Carcinoma; IE, In Evaluable; PR, Partial Remission; SD, Stable Disease; PD, Progressive Disease; DCR, Disease Control Rate.

Median PFS in the entire group of 33 patients was 22 weeks; it was 35 weeks among HCC patients and 14.5 weeks among BDC patients. Median OS time for the entire group was 28 weeks (7 months): 26 weeks among HCC patients and 33.5 weeks among BDC patients, as demonstrated in Table 6.

### Toxicities

Toxicities affecting more than 5% of the patients included fatigue (12.9%), neutropenia (9.6%), thrombocytopenia (9.6%), and diarrhea (6.4%). All grade 3 or greater treatment‐related toxicities are summarized in Table 7 below.

**Table 7:** Grade 3 or greater treatment‐related toxicities

Toxicity	Number	Percent (%)
Hematological		
Anemia (1 Hemolytic)	2	6
Neutropenia	7	21
Neutropenic fever	2	6
Thrombocytopenia	6	18
Gastrointestinal		
Nausea & vomiting	4	12
Diarrhea	4	12
Abdominal pain	5	15
Hepatic		
Bilirubin	4	12
Liver enzymes	5	15
Renal failure	1	3
Dermatitis (Acneiform)	3	9
Peripheral neuropathy	1	3
Metabolic	2	6
Cerebral hemorrhage due to combination of erlotinib and coumadin with INR >17	1 (Final)	3

## Discussion

Primary liver cancer which usually refers to hepatocellular carcinoma affects more than 500,000 individuals worldwide and as many as 20,000 patients per year in the US. Similarly, incidence of BDC has been steadily increasing in the US 1, 12. The treatment of HCC and of BDC has evolved over the last several years; thus, sorafenib has become the standard of care for the former, and the combination of gemcitabine and cisplatin for the latter 13, 14. However, the phase II trial reported here preceded the above‐mentioned regimens that have become standard therapy for HCC and BDC. Our protocol was launched in 2006, when no established standard therapy existed for HCC or for BDC. The combination of gemcitabine and oxaliplatin was based on publications by Louafi et al. 6. Since both HCC and BDC express epidermal growth factor receptors (EGFR), we hypothesized that EGFR is a driver of these diseases, and investigated a combination of chemotherapy with EGFR blockade in the form of the TKI erlotinib, based on previously reported erlotinib studies by Philip et al. 10, 11.

Initially, our trial was designed only for patients with HCC, however, following an incidental misdiagnosis of HCC in a patient who in reality had BDC, and the observation of an antitumor response, it was decided to expand eligibility to patients with both BDC and HCC. Only seven patients with BDC and 26 patients with HCC were accrued before the free supply of oxaliplatin was curtailed by the sponsor (Sanofi‐Aventis) requiring substitution of oxaliplatin by cisplatin which was covered by third party carriers. Consequently, this report covers only the patients treated with gemcitabine, oxaliplatin, and erlotinib with a diagnosis of HCC and BDC. Once the concomitant use of erlotinib and warfarin analogues was disallowed, the gemcitabine, oxaliplatin and erlotinib combination became quite well tolerated, and toxicities were manageable.

**Figure 11:**
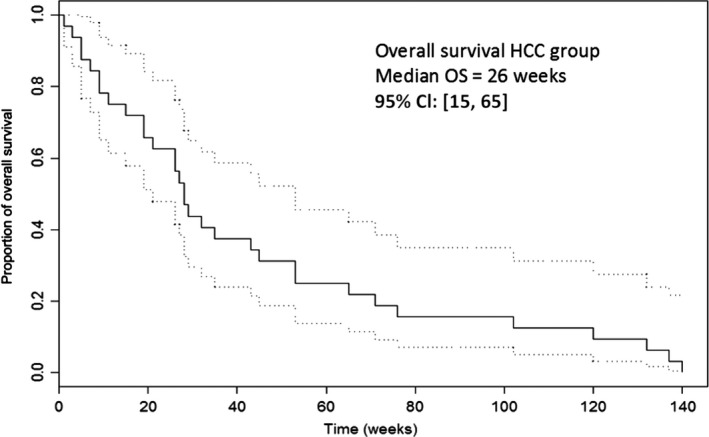
Overall Survival (OS) of 26 patients with Hepatocellular Carcinoma (HCC).

**Figure 12:**
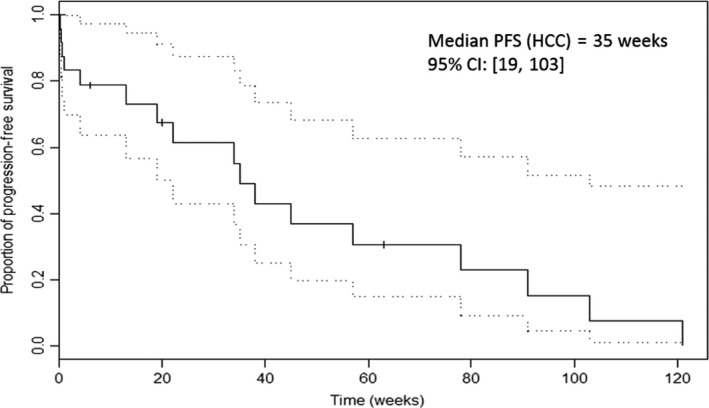
Progression‐Free Survival (PFS) of 26 patients with Hepatocellular Carcinoma (HCC).

Median progression‐free survival (PFS) for the entire group was 22 weeks (5.5 months) and median overall survival (OS) time for the entire group was 28 weeks (7 months). Median PFS and OS times among 26 HCC patients were 35 weeks (Fig. 11) and 26 weeks (Fig. 12), respectively. This phenomenon of a longer PFS than OS among HCC patients may be explained by the occasional death of patients due to complications from liver cirrhosis rather than from progression of the HCC. Thus, patients whose death could be clearly attributed to cirrhosis, namely end‐stage liver failure, hepato‐renal syndrome secondary to hypoalbuminemia, or death following severe hepatic encephalopathy, were censored at the time of their death.

**Figure 13:**
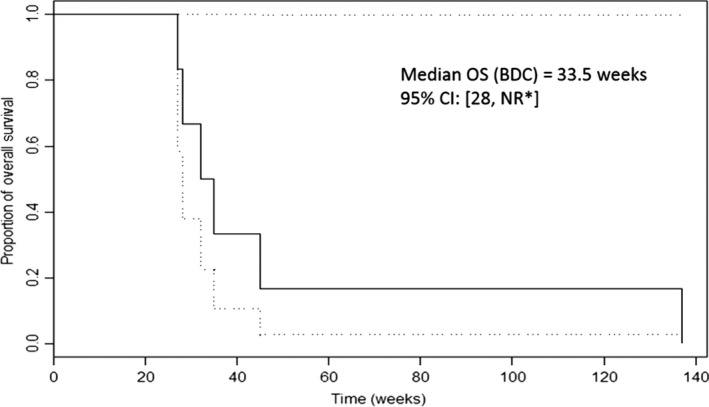
OS of seven patients with Biliary Duct Cancer (BDC).95% Confidence Interval of Median OS=[28, NR]. *No result (NR)

**Figure 14:**
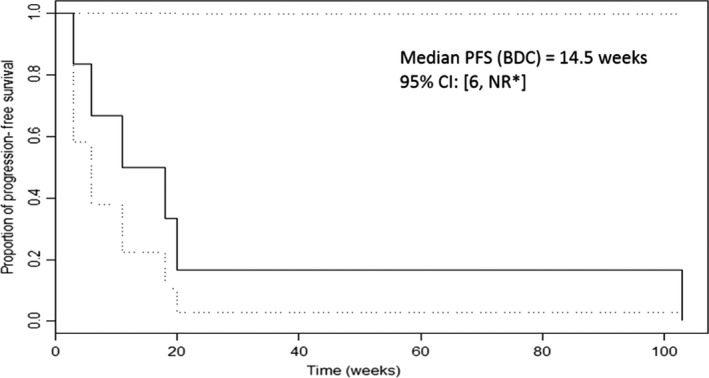
Progression‐Free Survival (PFS) among seven patients with BDC.Median PFS (BDC)=14.5 weeks 95% CI: 6, *No result (NR).

Among seven BDC patients, the respective median PFS and overall survival times (OS) were 14.5 weeks (Fig. 13) and 33.5 weeks (Fig. 14). The fact that the incidence and severity of liver cirrhosis were much lower among the seven patients with BDC can explain the difference in the discordance between HCC and BDC in terms of PFS and OS. Indeed, as indicated in Table 2, the incidence of HCV infection among HCC patients was 14/26 (54%), versus 0/7 (0%) among BDC patients. Moreover, among 2/7 BDC patients who had clinical evidence of liver cirrhosis it was related to ETOH abuse and not to HCV infection, a fact that may account for less severe manifestation of end stage liver disease. While the PFS and OS among HCC patients may have met the prespecified statistical endpoints but did not seem to impact the PFS and OS in a meaningful fashion as compared to reported results of gemcitabine‐oxaliplatin combination or erlotinib by itself, and therefore does not warrant recommending this regimen to patients with HCC 6, 10, 15.

In contrast, among patients with BDC, both PFS and OS seem encouraging enough to further investigate EGFR directed therapies in combination with chemotherapy and targeted therapies preferably in randomized fashion. Such study would likely have to be done with the combination of gemcitabine and cisplatin that has become the standard of care since the publication of the report by Valle et al. 14.

**Figure 15:**
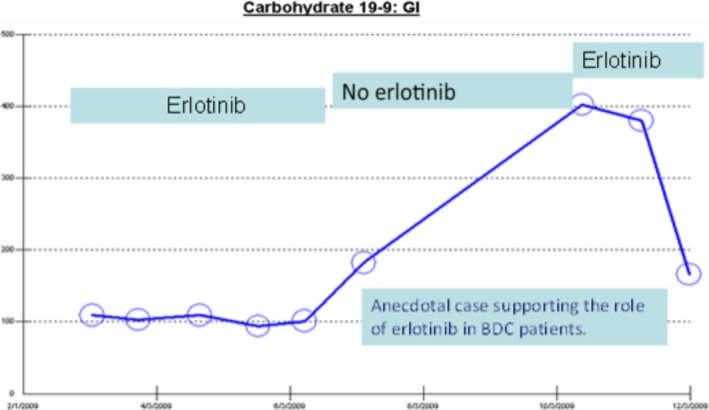
Patient with cholangiocarcinoma treated with Gem‐Ox, with and without erlotinib and his corresponding tumor marker CA19‐9.

Intriguing observations among BDC patients treated in this trial include two anecdotal cases. First one is of a 72‐year‐old male patient with metastatic BDC (intrahepatic cholangiocarcinoma) with bone metastasis, whose disease course is illustrated by CA‐19‐9 marker curve in Figure 15. He was started on the combination of gemcitabine, oxaliplatin, and erlotinib resulting in disease stabilization and improvement in performance status. Because of a pathological fracture of the left hip that required fixation of the femur followed by radiation therapy, protocol treatment was withheld for 5 weeks and the patient came off erlotinib treatment. This off label agent was initially not approved by the Medicare and the patient was given the chemotherapy without erlotinib, resulting in an increase in the tumor marker CA‐19‐9. Following an appeal, Medicare approved the off‐label use of the erlotinib for the patient. Once the erlotinib was resumed, antitumor and CA‐19‐9 biomarker responses were again observed and the patient continued to live with an excellent performance status for another 2 years before developing refractory disease. Our data have met the preset P0 and P1 stipulations of the primary objective of this study. However, we believe that our results do not have meaningful improvement over Gemcitabine + Oxaliplatin or erlotinib administered without chemotherapy, at least in HCC. The observation demonstrated in 2/7 patients with BDC warrants further studies of the EGFR‐targeted agents combination in BDC patients in biomarker selected population. A second case of a long‐term responder includes another patient with BDC cancer who presented with obstructive jaundice requiring bile duct decompression by ERCP, with measurable infiltrative disease surrounding the intrahepatic bile duct confluence (Klatskin tumor). The patient has had a remarkable PR, without any evidence of intrabiliary tumor and no further need for a stent and no clearly measurable disease on CT scan. The patient had subsequently recurrent disease that responded to gemcitabine, cisplatin, and erlotinib and later maintained on single agent erlotinib. Later disease recurrence has been treated with the combination of Gemciabine and nab‐Paclitaxel and erlotinib. The patient has been alive for more than 7 years. These are the only long‐term surviving patients enrolled in this trial to date. Although we did see two exceptional responders we acknowledge that size of patient population in BDC group in our study is small to draw conclusive survival analysis and is a limiting factor in this study**.**

Thus, based on our results although gemcitabine, oxaliplatin, and erlotinib combination has only modest benefit in HCC, BDC, however, might be a better patient population to study in future as we saw durable complete response in one patient and another patient had PR after addition of erlotinib. Lee et al. recently reported a phase III randomized trial results that looked at gemcitabine plus oxaliplatin with or without erlotinib in patients with metastatic biliary ductal, gall bladder and ampulla of vater cancer and were unable to show an overall survival advantage but interestingly an unplanned subgroup analysis showed statistically significant PFS advantage for erlotinib arm (5.9 vs. 3 months) in cholangiocarcinoma patients only 16. This study was conducted in South Korea with only Asian patient population. We have shown similar results in a small but diverse population in a US‐based. We believe a randomized phase II trial with this drug combination in patients with BDC is thus warranted in an occidental population. Interest in targeting EGFR in BDC is further strengthened by recent report by Goff et al. from Vanderbilt in which this group tested pulsatile erlotinib with oxaliplatin and gemcitabine for patients with advanced biliary tract patients and showed promising results 17 **.**

Another potential interest would be further molecular characterization of BDC patients as we saw long‐term responses in 2 out of 7 patients but their molecular status in regards to KRAS and EGFR status is not known to us. There have been a couple of attempts at targeting EGFR in cholangiocarcinoma with monoclonal antibodies instead of EGFR tyrosine kinase inhibitors. One such study (BINGO trial) did not show meaningful improvement in survival with cetuximab in combination with gemcitabine and cisplatin as compared to a placebo in cholangiocarcinoma patients, however, a more recent single arm trial (TACTIC trial) that included only KRAS wild type patients showed a response rate of 46% suggesting a more selected population as a better target 18, 19. We did not include HER1/EGFR expression analysis, however, studies performed with erlotinib prior to our study showed higher EGFR expression in BDC and HCC patients without correlation with erlotinib responsiveness and the study by Lee et al. subsequently also did not show beneficial predictive value of EGFR expression in patients being treated with this combination. A more recent subanalysis of this study looked at the molecular markers including KRAS, PIK3CA, and EGFR mutational status and benefit of adding erlotinib was statistically significant in KRAS wild type population. Based on this published data, KRAS mutant BDC patients are unlikely to respond to EGFR directed therapies and should be excluded from future EGFR directed studies, unless EGFR directed drugs are combined with other targeted therapies. As an example, recently published preclinical investigations of trametinib + panitumumab in BTC showed encouraging efficacy of combining these two agents 20. Unfortunately, we did not stratify patients by mutational status (EGFR, KRAS PIK3C) and our study was also limited by small number of patients and limited tissue availability in our patients.

The discontinuation of free supply of oxaliplatin for the study required switching to cisplatin, for which third party payment was easier to secure. Therefore, we continued this trial concentrating on BDC patients, and some patients were treated with gemcitabine, cisplatin, and erlotinib. The combination of gemcitabine + cisplatin is now the standard of care for the management of BDC. Therefore, we believe that EGFR‐directed therapies should be further explored in combination with other targeted therapies or with gemcitabine‐cisplatin in randomized trials in molecularly defined BDC patients.

## Conflict of Interest

Authors Yehuda Patt, Murad Waheed, Ari D Baron, Pranshu Bansal, Yanis Boumber, Ed Bedrick, Sang‐Joon Lee, Ruofei Du do not report any conflict of interest with this trial. Author Mohammad H. Fekrazad is on speaker bureau for Genentech and Amgen, Genentech provided the drug erlotinib for this trial.
